# UV Radiation and Protein Hydrolysates in Bio-Based Films: Impacts on Properties and Italian Salami Preservation

**DOI:** 10.3390/antiox13050517

**Published:** 2024-04-26

**Authors:** Viviane Patrícia Romani, Paola Chaves Martins, Meritaine da Rocha, Maria Carolina Salum Bulhosa, Felipe Kessler, Vilásia Guimarães Martins

**Affiliations:** 1 Laboratory of Food Technology, Federal University of Rio Grande, Rio Grande 96203-900, RS, Brazilvilasiamartins@furg.br (V.G.M.); 2 Federal Institute of Paraná—Pitanga Campus, Pitanga 85200-000, PR, Brazil; 3 Laboratory of Applied and Technological Physical Chemistry, Federal University of Rio Grande, Rio Grande 96203-900, RS, Brazil

**Keywords:** active packaging, antioxidant, film properties’ improvement, Italian salami

## Abstract

UV radiation was combined with the incorporation of fish protein hydrolysates to improve the performance of active bio-based films for food packaging. UV radiation was not used previously to enhance the packaging performance of blend films of starch/protein, and fish protein hydrolysates were not incorporated in bio-based polymer surfaces previously. Rice starch and fish proteins (from Whitemouth croaker muscle) were utilized to prepare films by the casting technique, which were UV-radiated under different exposure times (1, 5, and 10 min). The packaging performance of the films was determined according to the mechanical and barrier performance, solubility, and color. Fish protein hydrolysates (from Argentine croaker muscle) were then incorporated into the films (bulk structure or surface). The results showed that UV radiation for 1 min increased the tensile strength and modified the optical properties of films. It also altered the structure of the polymeric matrix, as demonstrated by the microstructure and thermal analysis, in agreement with the data obtained in packaging properties. The evaluation of antioxidant capacity through 2,2-azino-bis-3-ethylbenzthiazoline-6-sulphonic acid (ABTS) and reducing power indicated that incorporating fish protein hydrolysates either in the films’ bulk structure or film surface promoted antioxidant properties; control films (produced with rice starch/fish proteins without hydrolysates) also presented antioxidant potential. According to the peroxide value and thiobarbituric acid reactive substance (TBARS) assays, control films and the films containing hydrolysates in their bulk structure or on the surface could prevent the lipid oxidation of Italian salami. Thus, combining UV radiation to shape the characteristics of bio-based materials with fish protein hydrolysates to reduce lipid oxidation contributes to the performance of active bio-based films for food packaging.

## 1. Introduction

Protein hydrolysates are bioactive components generated from the breakage of protein molecules and might be obtained from foods, as well as food industry residues/byproducts [1,2]. Fish have been considered an important source of protein hydrolysates due to their antioxidant capacity and potential to inhibit lipid oxidation [3,4]. Argentine croaker (*Umbrina canosai*) is an underutilized fish and has low commercial value, making it a promising raw material for hydrolysates obtaining. Indeed, its protein hydrolysates presented antioxidant, antimicrobial, and anti-inflammatory properties in previous studies [5]. Among the potential applications of fish protein hydrolysates is the production of bioactive materials for food packaging. They can be incorporated either in the bulk polymer structure or on the polymer surface, providing functionality to the matrices. Such materials are called active packaging, which expands food shelf life and reduces waste. Active polymers might prevent the growth of pathogenic/deteriorating microorganisms, and/or decrease deterioration reactions initiated by oxygen, such as lipid oxidation [6,7,8]. Active packaging technologies are applied to a wide variety of foods, including dairy, meat products, fruits, and vegetables [9]. Italian salami is a potential product to be packaged with antioxidant films containing fish protein hydrolysates. Its composition has up to 30% of lipids, which are prone to oxidize [10].

Besides active compounds, food industry wastes and byproducts with low commercial value are also sources of biomacromolecules such as proteins, polysaccharides, and lipids. They are promising raw materials to obtain environmentally friendly polymer alternatives to synthetic plastics [11,12]. Biodegradable packaging, made from renewable natural polymers, emerged to mitigate the environmental impact caused by petroleum-based polymers [13,14]. Up to now, a wide variety of bio-based films have been produced using different molecules and bioactive substances. For example, biomacromolecules such as starch and fish proteins from low-value resources were used in the development of blends incorporated with natural compounds [15,16,17]. Even though promising results have been reported, one of the hurdles for their commercial application is the low packaging performance, mainly caused by their hydrophilic nature [18,19].

In terms of polymer improvement, radiation technologies are ecological alternatives to enhance the performance of materials. The exposure of polymers to UV radiation treatment, for example, can cause oxidation, cross-linking, and/or degradation of the polymer structure. It works by creating free radicals, which interact with the material’s surface changing its characteristics [20,21]. Rezaee et al. [22]; Fathi, Almasi, and Pirouzifard [23]; and Schmid et al. [24] previously observed changes in bio-based film properties treated with UV radiation. Additionally, this technology can contribute to improving active materials efficacy. This occurs when UV radiation is used to incorporate functional components on the material’s surface instead of adding bioactive compounds to the bulk polymer structure [25]. This approach is known as advantageous, as the components do not need to diffuse from the bulk structure to the surface to provide functionality, and the risk of losing the biomolecule activity is reduced [26,27].

To the best of our knowledge, UV radiation has not been used previously to enhance the packaging performance of blend films of starch/protein, and fish protein hydrolysates have not been incorporated in bio-based polymers’ surfaces. Thus, this study aimed (i) to combine UV radiation and fish protein hydrolysates to (i) increase the packaging performance of blend films of starch/protein; (ii) identify the most promising way of incorporating protein hydrolysates (bulk structure or surface) in film matrices; and (iii) confirm their ability to prevent the lipid oxidation of sliced Italian salami. UV radiation improved the packaging characteristics of rice starch/fish protein films. In addition, all the films showed the potential to increase the shelf life of high lipid content products, as observed in the results presented for Italian salami.

## 2. Materials and Methods

### 2.1. Materials

Arrozeira Pelotas (Pelotas, RS, Brazil) was the supplier of broken rice grains (*Oryza Sativa* L.) for starch extraction. The fish species Whitemouth croaker (*Micropogonias furnieri*) and Argentine croaker (*Umbrina canosai*) were acquired from local processing industries (Rio Grande, RS, Brazil). The biomolecules of starch from rice and proteins from the Whitemouth croaker were extracted as described previously [16]. The enzyme Alcalase^®^ was supplied by Novozymes Latin America (Araucária, Brazil). Sliced Italian salami was obtained in a local market (Rio Grande, RS, Brazil). Polypropylene bags were from Labplas (Sainte-Julie, QC, Canada). All the chemicals employed in the assays were of analytical grade.

### 2.2. Protein Hydrolysate Preparation

Fish protein hydrolysates were generated from protein isolate that was obtained with the pH-shifting method using Argentine croaker muscle [5]. The hydrolysis of the protein isolate was carried out according to Raghavan and Kristinsson [28] utilizing the enzyme Alcalase. It was added in a 30 U/g enzyme/protein ratio, based on the enzyme activity (13.3 U/mg protein). The hydrolysis was conducted until reaching the degree of 20%, determined using the pH-stat method [29]. The enzyme was then inactivated, and the sample was centrifuged. Lastly, the supernatant containing the hydrolysates was lyophilized and stored at −18 °C until its use.

### 2.3. Film Production and UV Radiation Treatment

Starch/protein blend films were produced using the casting technique according to Romani et al. [16]. Briefly, rice starch and fish proteins (3%) were homogenized in water at the ratio of 25/75 with glycerol (20%) as the plasticizer. pH was adjusted to 11.0, and the mixture was kept agitating at 80 °C for 20 min. After cooling, the film-forming solution was placed in Petri dishes and dried at 40 °C. The films were stored in desiccators (50% relative humidity) for 24 h before UV radiation treatment.

UV radiation was applied at room temperature (23 ± 1 °C) in control films (without protein hydrolysates) using a procedure adapted from previous studies [30,31]. Constant irradiation was provided with a medium-pressure mercury lamp (400 W) with films placed 23 cm from the lamp. The wavelengths were 250–400 nm, and the radiation dose was 3.67 × 10^17^ photons/min.cm^2^. The radiation effects were studied at 1, 5, and 10 min. The most appropriate time for UV radiation treatment of the films was chosen based on the packaging performance results, which were evaluated through physicochemical analysis (described below). Films that were irradiated according to the most appropriate time were also characterized by their microstructure characteristics and thermal properties.

For the films containing fish protein hydrolysates in their bulk structure, protein hydrolysates were added to the starch/protein solution in a 5% (*w*/*v*) concentration after cooling to room temperature and were then homogenized and placed in Petri dishes. After drying, they remained for 24 h in desiccators, and then they were UV-treated for 1 min. For the films containing fish protein hydrolysates on the surface, 1.0 mL of the aqueous protein hydrolysate solution (5%, *w*/*v*) was spread on the film’s surface after UV treatment for 1 min and then dried at 40 °C. Antioxidant properties and the film’s capacity to prevent the lipid oxidation of sliced Italian salami were assessed for control films (without protein hydrolysates), films containing protein hydrolysates in their bulk structure, and surface-coated films.

### 2.4. Film Evaluation

#### 2.4.1. Packaging Characteristics

The thickness of the films was evaluated in 10 different positions using a micrometer (Insize, IP54, Boituva, Brazil). Mechanical properties (tensile strength and elongation at break) were measured following the ASTM D882-02 method [32] in a texture analyzer (TA.XTplus, Stable Micro Systems, Surrey, UK). Solubility in water (SW) was determined considering the weight loss after the immersion of dried film discs in water for 24 h [33]. Water vapor permeability (WVP) was assessed using ASTM E96-00 [34] based on the weight gain of permeation cells containing films sealed on top and silica (desiccant) inside. Color properties were measured with the CIELab color space system using a colorimeter (Minolta Chroma Meter, CR-400, Osaka, Japan). The results were expressed as the total difference in color (ΔE*), as described by Filipini et al. [33]. Opacity (%) was also determined using the colorimeter and calculated as the relationship between the value obtained from the film superimposed on a black and a white standard.

#### 2.4.2. Microstructure and Thermal Properties

The film’s surface characteristics were studied by morphology observation through scanning electron microscopy (SEM) with a Jeol, JSM-6060 microscope. Energy-dispersive X-ray spectroscopy (EDX) was performed to determine the element’s concentration on the film’s surface under 10 kV incident electron energy. X-ray diffraction (XRD) was performed to evaluate the crystalline areas using a Bruker D8 Advance diffractometer under a voltage of 40 kV, current of 30 mA, and an incident angle of 2Ɵ (10 to 50°). The measurements of attenuated total reflection Fourier transform infrared spectroscopy (ATR-FTIR) were performed using a Prestige 21 model (Shimadzu) equipment, and the spectra were obtained under the spectral resolution of 4 cm^−1^. A calorimeter (Shimadzu, TGA-60, Kyoto, Japan) with a nitrogen flow rate of 50 mL/min was utilized to determine the thermal properties through differential scanning calorimetry (DSC). For DSC, samples were sealed in aluminum pans and submitted to the heating rate of 10 °C/min to be scanned from 30 to 200 °C.

#### 2.4.3. Antioxidant Properties of Films

To perform the antioxidant assays, film samples (0.1 g) were incubated with 10 mL of 200 mM phosphate buffer (pH 6.6), stirring overnight [35]. Films without protein hydrolysates (control) and samples containing protein hydrolysates either in the bulk polymer structure or on the polymer surface were evaluated for radical scavenging capacity using 2,20-azino-bis-3-ethylbenzthiazoline-6-sulphonic acid (ABTS) and reducing power. ABTS assay was performed as described by Bitencourt et al. [36], with an absorbance reading at 734 nm using a microplate reader (Celer, Polaris, Belo Horizonte, Brazil). Reducing power was determined based on the study by Canabady-Rochelle et al. [37]. The absorbances were read at 700 nm, where higher absorbance indicated a higher reducing power.

### 2.5. Film Evaluation

The effectiveness of the films in preventing lipid oxidation was determined using sliced Italian salami as a model due to the lipid content, which is prone to oxidization. The films were placed among the salami slices (Figure S1) and stored in polypropylene polymer bags for 21 days at 15 ± 1 °C to accelerate lipid oxidation. Lipid oxidation was measured in 0, 3, 7, 14, and 21 days by the peroxide value (PV) and thiobarbituric acid reactive substances (TBARSs). Control salami samples (without films), samples packaged with control films (starch/protein blend without protein hydrolysates), and samples containing protein hydrolysates in the bulk composition or on the surface were analyzed.

PV was determined using lipids extracted with a water–methanol–chloroform solution [38]. Acid–chloroform, saturated KI, and distilled water were added to the lipid samples and titrated using a 0.1 N sodium thiosulfate solution. The results were expressed as milliequivalents (mEq) of active oxygen per kilogram of lipid [39]. TBARSs were determined according to Song et al. [40], with modifications. In summary, salami samples were homogenized with trichloroacetic acid. After centrifugation, the supernatant was mixed with TBARS reagent and immersed in a boiling water bath at 95 °C for 45 min. After cooling, the absorbance was measured at 538 nm (Kazuaki, IL-592, Wuxi, China). A standard curve of malonaldehyde using 1,1,3,3-tetraethoxypropane (TEP), a precursor of MDA, was plotted, and the results were expressed as mg malonaldehyde/kg sample.

### 2.6. Statistical Analysis

All analyses were performed in at least triplicate, and standard deviations were calculated. Statistica 7.0 was used to perform an analysis of variance (ANOVA), and the means were compared by Tukey’s test, considering the values significantly different at *p* < 0.05.

## 3. Results and Discussion

### 3.1. Packaging Characteristics of UV-Radiated Films

The physicochemical properties of packaging materials drive their application in foods since each type of product demands a specific performance. Table 1 demonstrates the results obtained for the control films and UV-treated films. The thickness of films was significantly similar (*p* > 0.05) for the different times studied. However, UV radiation significantly increased (*p* < 0.05) the tensile strength and decreased elongation at the break of the films. These modifications probably occurred because of chemical reactions caused by the absorption of electromagnetic energy from UV radiation. It may have promoted the formation of free radicals in fish proteins, making them reactive to establish interactions with other protein chains and amylopectin/amylopectin from starch. Other authors reported the same trend in UV-radiated films. Gennadios et al. [41], for example, attributed the superior tensile strength and the lower elasticity in soy protein films treated with UV radiation to the process of cross-linking in the structure of the films, making them stronger and less extensible. Khan et al. [42] observed similar behavior in starch films exposed to UV radiation, which led to an increase of around 1 to 1.5 MPa in tensile strength after UV treatment. They explained the cross-linking formed by the UV treatment since radiation can degrade some starch chains and increase the hydrogen bonds among the molecules. Ustunol and Mert [43] also reported that changes in the mechanical performance of whey protein films after UV treatment can be attributed to the double bonds and aromatic rings present in protein molecules, which absorb the energy. This energy promotes the formation of free radicals that generate new intra- and inter-molecular bonds within polymer chains. These authors observed an increase in tensile strength of around 2 MPa after UV treatment, while in the present study, an increase of approximately 4 to 5 MPa was observed. In contrast, they obtained a 4% decrease in elongation, while a 24–30% decrease was recorded in the present study.

Water vapor permeability and solubility were significantly similar (*p* > 0.05) for films without UV treatment and UV-radiated films (Table 1). The same trend was reported by Díaz et al. [44] and Schmid et al. [24], applying UV treatment in whey protein matrices. Other authors [45] also mentioned that the hydrophilic behavior of polymers may not be highly affected by the UV treatment. They explained that extensive cross-linking is necessary to extensively change the WVP of films.

The total color difference (ΔE*) increased significantly (*p* < 0.05) after 5 and 10 min of UV radiation, while the opacity of the films differed significantly (*p* < 0.05) for all samples. Schmid et al. [24] had similar results for whey protein films treated with UV radiation and indicated changes in the aromatic rings and the possible formation of photo composites. Additionally, variations in ΔE* were explained by the ability of UV radiation to promote the formation of conjugated double bonds in protein molecules, which increased the intensity of yellow coloration.

Based on these results, the most appropriate time of exposure of the starch/protein films to UV radiation was 1 min. This time of treatment did not change solubility and WVP; however, it increased tensile strength, keeping some flexibility of the film (important for most food packaging applications). Then, as it was considered superior to the other treatment times, it was used for further analysis of the films in terms of their microstructure, antioxidant properties, and ability to prevent the lipid oxidation of sliced Italian salami.

### 3.2. Film Microstructure and Thermal Properties

**Figure 1:**
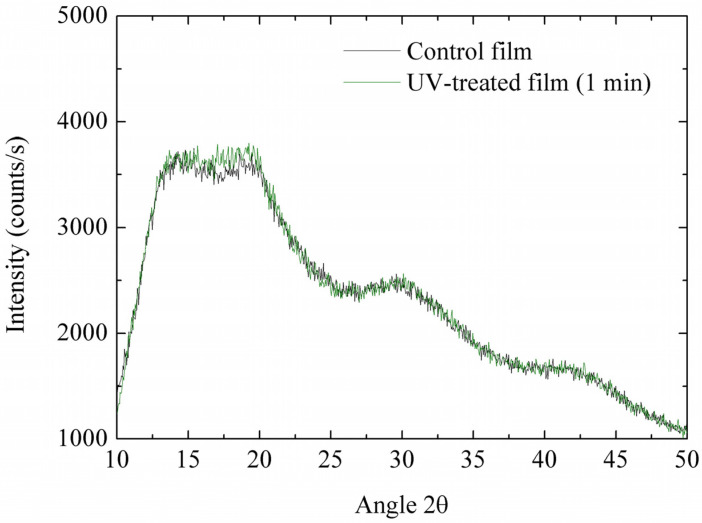
Effects of UV radiation treatment in the X-ray diffraction pattern of starch/protein films.

X-ray diffraction (Figure 1) patterns presented mainly amorphous structures, typical of the molecules used. Four main diffraction peaks, centered around 2θ = 14.5°, 2θ = 19°, 2θ = 29.6°, and 2θ = 41.5°, were present. They have distances of 6.15 Å, 4.73 Å, 3.12 Å, and 2.32 Å, respectively, which are characteristic of protein molecules [46]. Besides that, rice starch is known to have the A-type XRD pattern, with strong reflection at 16.8, 18.0, and 22.7° [47]. Minor changes occurred after 1 min of UV radiation, even in the relative crystallinity (13.8% for control film and 14.7% for UV-treated film), in accordance with the results of physicochemical characterization, which were similar. Slightly higher crystallinity agrees with superior strength and lower flexibility, as previously observed [48]. Khan et al. [42] also observed that the degree of crystallinity after the UV treatment of films remained similar.

The morphology of films showed predominantly smooth and homogeneous surfaces for control (no UV) and treated films (Figure S2). The incorporation of UV radiation/protein hydrolysates caused a slightly rougher surface with small protrusions. Cross-section images showed a compact structure over the film matrices in both control and treated samples. Such observations indicate that UV radiation treatment did not penetrate enough in the matrix structure to cause significant modification in film properties, as also observed by Fathi et al. [23]. Overall, the image observations are in accordance with the XRD pattern, where minor differences were presented. The morphology of films in the present study was different from the study by Díaz et al. [44], who observed irregular agglomerates, discontinuous areas similar to fractures, and large smooth structures after UV treatment, attributed to denaturation and increase of bonds between proteins. The concentration (%) of elements of the control films analysis showed a higher amount of oxygen (52.2%), followed by carbon (25.0%) and nitrogen (17.4%), typical of films’ macromolecules (starch and protein). The incorporation of UV radiation and protein hydrolysates increased carbon (26.5%) and nitrogen (17.9%) and decreased oxygen (50.3%) contents, which were characteristic of the protein hydrolysates incorporated on the film’s surface.

**Figure 2:**
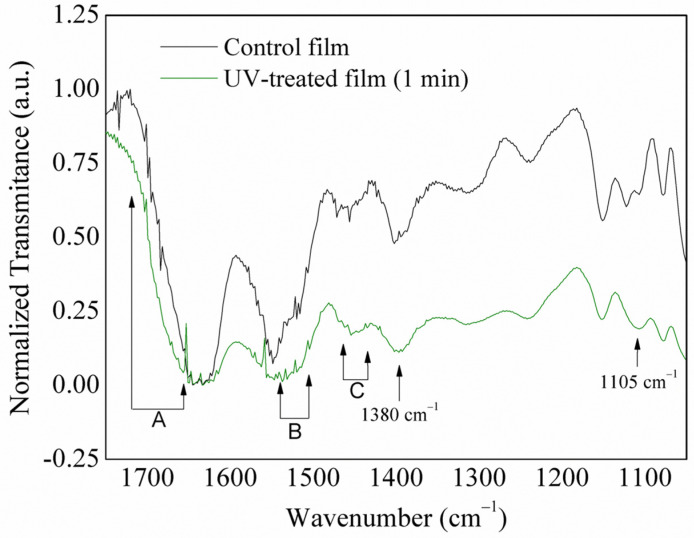
ATR-FTIR spectra of control (no UV) and UV-treated (1 min) starch/protein films coated with protein hydrolysates.

The ATR-FTIR spectra of untreated films (Figure 2) showed the main bands of proteins as C-N stretching (1550 cm^−1^), C=O stretching (1640 to 1690 cm^−1^), C-O stretching (1440 and 1480 cm^−1^), and C-C stretching (1390 cm^−1^). These bands are associated with the fish protein used for film production and the coating of protein hydrolysates on the films’ surface. It was observed that UV radiation and coating with protein hydrolysates modified the surface. The UV treatment in the presence of oxygen mostly indicated the inclusion of chemical groups. The region indicated as ‘A’ showed an increase in signal, characteristic of C=O stretching (1670 to 1720 cm^−1^). The band assigned to the -N stretching (1550–1500 cm^−1^), region ‘B’, appeared after UV radiation. Region ‘C’ also indicated an increase in C-O-C signals and an increase in C-C stretching signal in 1380 cm^−1^ and C-H signal in 1105 cm^−1^. These are typical FTIR signals of protein hydrolysates [49,50].

DSC thermograms showed endothermic peaks for all samples. UV radiation modified the glass transition temperature (T_g_), melting temperature (T_m_), and enthalpy (∆H) of the films (Table S1). Such modifications suggest changes in the mobility of chains on the films’ structure. Protein hydrolysates have low molecular weight, and when incorporated into the surface of the film, they might have caused a decrease in T_g_ and T_m_ [51]. Ustunol and Mert [43] also observed a decrease in T_g_ after UV radiation was used for the cross-linking of whey protein films.

### 3.3. Antioxidant Properties of Films Incorporated with Protein Hydrolysates

UV-treated films containing protein hydrolysates in the bulk structure or on the surface, as well as control films (no hydrolysate incorporation) were studied by their radical scavenging and reducing power capacity. As shown in Table 2, even control films presented antioxidant activity in both assays. This is probably due to the capacity of fish proteins to act as electron donors and free radical scavengers. Proteins from the Whitemouth croaker, used in the films’ production, were previously studied and showed 55.7% of hydrophobic amino acids, which are associated with the antioxidant activity of fish proteins [52,53].

The incorporation of protein hydrolysates either in the film bulk structure or on the surface significantly increased (*p* < 0.05) the antioxidant activity, as demonstrated by ABTS and reducing power assays, confirming the positive effect of their use as antioxidant agents. According to Rocha et al. [5], the antioxidant activity of hydrolysates from Argentine croaker is attributed to their low molecular weight distribution (1083 Da) and the elevated quantity of hydrophobic amino acids (365.44 mg/g—Table S2). In addition, reactive species can be stabilized by aromatic amino acids by transferring protons/electrons, preserving their stability through resonance structures [54]. Such amino acids were present in protein hydrolysates (77.00 mg/g—Table S2), contributing to the observed results. Thus, the use of protein hydrolysates as antioxidant agents in films could be effective in inhibiting lipid oxidation because of their capacity to donate electrons and/or scavenge radicals. The findings of the present study reveal that control films and films incorporated with protein hydrolysates have similar or superior antioxidant capacity compared with other materials reported in the literature, such as clary sage oil [55], phycocyanin [56], grapefruit essential oil [57], and procyanidins [58].

The incorporation of protein hydrolysates on the film surface resulted in a significantly lower (*p* < 0.05) antioxidant activity in comparison to the incorporation into the bulk structure, according to the reducing power test. This might have resulted from the interactions of hydrolysates with the film structure, which are different when added to the bulk composition or coated on the surface of the polymer. Furthermore, UV radiation changes the surface of polymers through different reactions, including the creation of free radicals; they could make the bonds with protein hydrolysates more stable, consequently decreasing their capacity to donate protons/electrons. Therefore, regardless of the way protein hydrolysates were incorporated into the starch/protein blends (composition or surface), the results presented by the antioxidant property assays indicated the possibility of these materials being applied as active packaging.

### 3.4. Films’ Effectiveness in Preventing Lipid Oxidation

**Figure 3:**
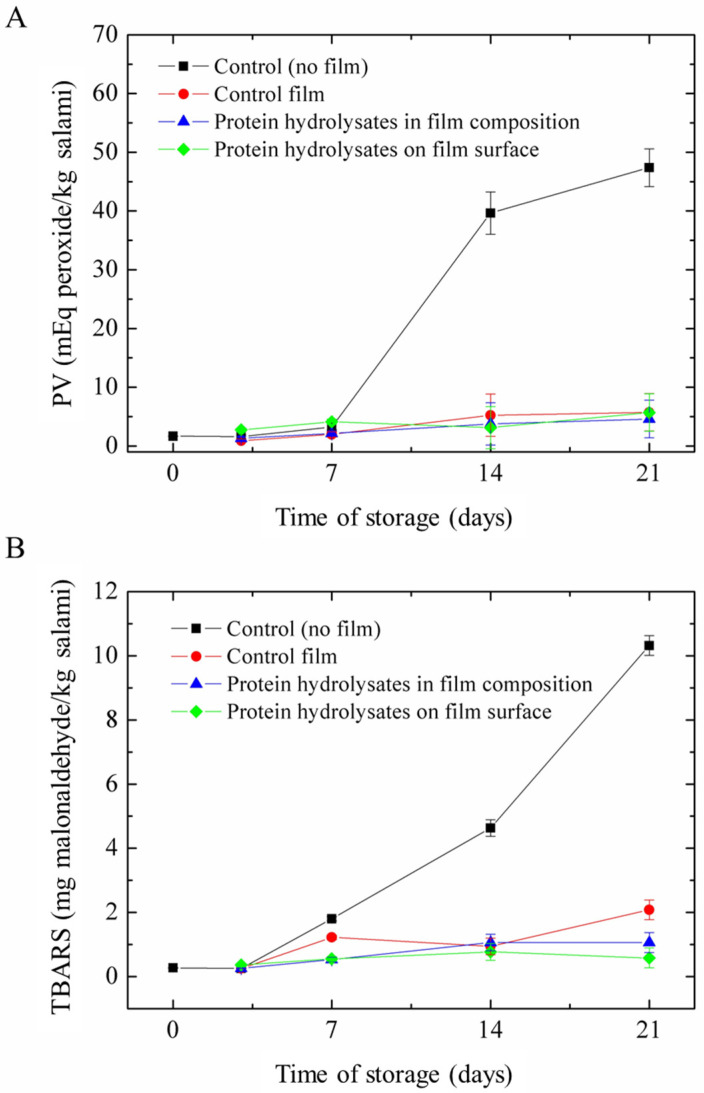
Effects of active films in (**A**) peroxide value (PV) and (**B**) thiobarbituric acid reactive substances (TBARS) of Italian salami. Means of three replicates ± standard deviations are presented.

All the films tested had the capacity to prevent lipid oxidation, as demonstrated by the lower rise in PV and TBARSs from 7 to 21 days compared to the salami samples without films (control) (Figure 3). In general, PV and TBARSs were similar in the three film formulations tested (control, protein hydrolysates in composition, and protein hydrolysates on the surface), in line with the observations of antioxidant properties (Table 2) and confirming the functionality of fish proteins used in film production. This behavior may have occurred due to the existence of hydrophobic amino acids in protein sequences, as mentioned previously, which were soluble in the lipids of the sample. Barbosa-Pereira et al. [59] obtained higher lipid oxidation in beef when using antioxidant films containing natural extract obtained from brewery residual waste and a commercial rosemary extract. TBARS values in the 9 days under analysis were superior to those reported in the present study. However, Thakur et al. [60] reported similar results when incorporating curcuma hydroethanolic extract in gelatin/chitosan lactate and applying it to chicken meat. However, it is important to consider that, in this case, experiments were performed at 4 °C, which slowed the lipid oxidation rate.

In 21 days, TBARS values (Figure 3B) were higher for salami samples with the control film compared to the films incorporated with protein hydrolysates, regardless of the way of incorporation. This observation indicates that the incorporation of fish protein hydrolysates in the films is advantageous to increase the functionality of the matrices. Besides that, the incorporation of protein hydrolysates on the film’s surface resulted in lower TBARS values in comparison to the incorporation in film composition. Even though there was a slight difference, the results confirm that the incorporation of hydrolysates on the film surface results in superior performance compared to the addition of film composition. However, even though there was a difference between the treatments, they presented an overall similar performance, suggesting that the UV treatment applied after film drying or before hydrolysate incorporation did not significantly affect the active properties of the films.

Consumers might identify off-flavors or off-taste in products when TBARS concentrations are equal to or higher than 5 mg malonaldehyde/kg [61]. All the films were effective in preventing salami from reaching that amount over the time studied. The findings of the lipid oxidation tests using Italian salami indicate that the films developed in this study, even the control films, are promising for application as active polymers in food packaging.

## 4. Conclusions

The use of UV radiation to enhance the packaging performance of blend films of starch/protein combined with the incorporation of fish protein hydrolysates in bio-based polymer surfaces was not reported previously. UV radiation enhanced the packaging characteristics of rice starch/fish protein films. It was found that it caused an increase in tensile strength and a decrease in elongation at the break of films. Color properties were also affected by the application of UV radiation, while solubility and WVP were not modified by the times tested. One minute of UV radiation was the most adequate time, especially because of the increase in tensile strength. Antioxidant activity results demonstrated that even the control films (without protein hydrolysates) are promising active packaging materials due to the amino acid composition of the proteins used. The incorporation of protein hydrolysates promoted a slight increase in antioxidant activity. Control films (without hydrolysates) and the films containing hydrolysates in the bulk structure or on the surface showed the potential to expand the shelf life of high-lipid-content products, as demonstrated by the decrease in the lipid oxidation of Italian salami, mainly when hydrolysates were added to the film surface. Combining UV radiation to develop the characteristics of films with the incorporation of fish protein hydrolysates to reduce lipid oxidation contributes to the performance of active bio-based films for food packaging. The use of these films is promising to reduce the deterioration of foods by decreasing oxidation reactions and because of their sustainable nature (aligned to the current demands of environmentally friendly alternative polymers).

## Figures and Tables

**Table 1:** Physicochemical properties of control UV-radiated films under different times of exposure.

Properties	No UV	UV 1 min	UV 5 min	UV 10 min
Thickness (mm)	0.081 ± 0.02 ^a^	0.074 ± 0.03 ^a^	0.084 ± 0.06 ^a^	0.082 ± 0.04 ^a^
Tensile strength (MPa)	13.85 ± 0.05 ^b^	18.31 ± 0.96 ^a^	16.26 ± 0.64 ^ab^	17.24 ± 1.49 ^a^
Elongation at break (%)	35.4 ± 2.1 ^a^	11.0 ± 0.6 ^b^	5.0 ± 0.1 ^c^	5.2 ± 0.5 ^c^
Water vapor permeability (g.mm/day.m^2^.kPa)	7.47 ± 0.84 ^a^	7.23 ± 0.18 ^a^	7.37 ± 0.21 ^a^	6.61 ± 0.27 ^a^
Solubility in water (%)	20.8 ± 0.4 ^a^	21.6 ± 1.0 ^a^	24.0 ± 0.6 ^a^	22.1 ± 2.0 ^a^
Total difference in color (ΔE*)	5.44 ± 0.33 ^b^	5.46 ± 0.21 ^b^	6.57 ± 0.11 ^a^	7.04 ± 0.34 ^a^
Opacity (%)	10.7 ± 0.2 ^b^	11.3 ± 0.0 ^a^	11.5 ± 0.2 ^a^	11.2 ± 0.2 ^a^

Means of at least three replicates ± standard deviations. Means in the same line with different superscript letters are significantly different (*p* < 0.05).

**Table 2:** Antioxidant properties of control films and films incorporated with protein hydrolysates.

Film Sample	ABTS Assay (%)	Reducing Power (700 nm)
Control (no hydrolysates)	64.8 ± 0.2 ^b^	0.257 ± 0.004 ^c^
Protein hydrolysates in composition	77.1 ± 0.5 ^a^	0.378 ± 0.011 ^a^
Protein hydrolysates on the surface	78.3 ± 0.6 ^a^	0.331 ± 0.005 ^b^

Means of three replicates ± standard deviations. Means in the same line with different superscript letters are significantly different (*p* < 0.05).

## Data Availability

The raw data supporting the conclusions of this article will be made available by the authors upon request.

## Supplementary Materials

The following supporting information can be downloaded at: https://www.mdpi.com/article/10.3390/antiox13050517/s1, Figure S1. Lipid oxidation assay of films containing sliced Italian salami; Figure S2. Effects of UV-light treatment on surface morphology (1) and cross-section area (2) of starch/protein films: (a) control film and (b) UV-treated film (1 min) coated with protein hydrolysates; Table S1. DSC characterization of control and UV-radiated films coated with protein hydrolysates; Table S2. Amino acid composition of protein hydrolysates. Reference [62] is cited in the Supplementary Materials.

## References

[1] Singh N. (2022). Proteins Isolates and Hydrolysates: Structure-function Relation, Production, Bioactivities and Applications for Traditional and Modern High Nutritional Value-added Food Products. Int. J. Food Sci. Technol..

[2] López-Moreno M., Jiménez-Moreno E., Márquez Gallego A., Vera Pasamontes G., Uranga Ocio J.A., Garcés-Rimón M., Miguel-Castro M. (2023). Red Quinoa Hydrolysates with Antioxidant Properties Improve Cardiovascular Health in Spontaneously Hypertensive Rats. Antioxidants.

[3] Riolo K., Rotondo A., La Torre G.L., Marino Y., Franco G.A., Crupi R., Fusco R., Di Paola R., Oliva S., De Marco G. (2023). Cytoprotective and Antioxidant Effects of Hydrolysates from Black Soldier Fly (*Hermetia illucens*). Antioxidants.

[4] García-Moreno P.J., Batista I., Pires C., Bandarra N.M., Espejo-Carpio F.J., Guadix A., Guadix E.M. (2014). Antioxidant Activity of Protein Hydrolysates Obtained from Discarded Mediterranean Fish Species. Food Res. Int..

[5] Da Rocha M., Alemán A., Baccan G.C., López-Caballero M.E., Gómez-Guillén C., Montero P., Prentice C. (2018). Anti-Inflammatory, Antioxidant, and Antimicrobial Effects of Underutilized Fish Protein Hydrolysate. J. Aquat. Food Prod. Technol..

[6] López-Cano A.A., Martínez-Aguilar V., Peña-Juárez M.G., López-Esparza R., Delgado-Alvarado E., Gutiérrez-Castañeda E.J., Del Angel-Monroy M., Pérez E., Herrera-May A.L., Gonzalez-Calderon J.A. (2023). Chemically Modified Nanoparticles for Enhanced Antioxidant and Antimicrobial Properties with Cinnamon Essential Oil. Antioxidants.

[7] Rathod N.B., Bangar S.P., Šimat V., Ozogul F. (2023). Chitosan and Gelatine Biopolymer-based Active/Biodegradable Packaging for the Preservation of Fish and Fishery Products. Int. J. Food Sci. Technol..

[8] Flórez M., Cazón P., Vázquez M. (2023). Characterization of Active Films of Chitosan Containing Nettle Urtica Dioica L. Extract: Spectral and Water Properties, Microstructure, and Antioxidant Activity. Int. J. Biol. Macromol..

[9] Wyrwa J., Barska A. (2017). Innovations in the Food Packaging Market: Active Packaging. Eur. Food Res. Technol..

[10] Vilarinho F., Andrade M., Buonocore G.G., Stanzione M., Vaz M.F., Sanches Silva A. (2018). Monitoring Lipid Oxidation in a Processed Meat Product Packaged with Nanocomposite Poly(Lactic Acid) Film. Eur. Polym. J..

[11] Zhang Y., Simpson B.K., Dumont M.-J. (2018). Effect of Beeswax and Carnauba Wax Addition on Properties of Gelatin Films: A Comparative Study. Food Biosci..

[12] Zhang W., Liu J., Zhang T., Teng B. (2024). A High-Performance Food Package Material Prepared by the Synergistic Crosslinking of Gelatin with Polyphenol–Titanium Complexes. Antioxidants.

[13] Lopes A.C., Klosowski A.B., Carvalho B.M., Olivato J.B. (2022). Application and Characterisation of Industrial Brewing By-products in Biodegradable Starch-based Expanded Composites. Int. J. Food Sci. Technol..

[14] Chaari M., Elhadef K., Akermi S., Ben Akacha B., Fourati M., Chakchouk Mtibaa A., Ennouri M., Sarkar T., Shariati M.A., Rebezov M. (2022). Novel Active Food Packaging Films Based on Gelatin-Sodium Alginate Containing Beetroot Peel Extract. Antioxidants.

[15] Peerzada Gh J., Sinclair B.J., Perinbarajan G.K., Dutta R., Shekhawat R., Saikia N., Chidambaram R., Mossa A.-T. (2023). An Overview on Smart and Active Edible Coatings: Safety and Regulations. Eur. Food Res. Technol..

[16] Romani V.P., Prentice-Hernández C., Martins V.G. (2017). Active and Sustainable Materials from Rice Starch, Fish Protein and Oregano Essential Oil for Food Packaging. Ind. Crops Prod..

[17] Romani V.P., Hernández C.P., Martins V.G. (2018). Pink Pepper Phenolic Compounds Incorporation in Starch/Protein Blends and Its Potential to Inhibit Apple Browning. Food Packag. Shelf Life.

[18] Nastasi J.R., Fitzgerald M.A., Kontogiorgos V. (2023). Tuning the Mechanical Properties of Pectin Films with Polyphenol-Rich Plant Extracts. Int. J. Biol. Macromol..

[19] Romani V.P., Olsen B., Pinto Collares M., Meireles Oliveira J.R., Prentice C., Guimarães Martins V. (2019). Plasma Technology as a Tool to Decrease the Sensitivity to Water of Fish Protein Films for Food Packaging. Food Hydrocoll..

[20] Drobota M., Gradinaru L.M., Ciobanu C., Stoica I. (2015). Collagen Immobilization on Poly(Ethylene Terephthalate) and Polyurethane Films after UV Functionalization. J. Adhes. Sci. Technol..

[21] Kowalonek J. (2017). Studies of Chitosan/Pectin Complexes Exposed to UV Radiation. Int. J. Biol. Macromol..

[22] Rezaee M., Askari G., EmamDjomeh Z., Salami M. (2020). UV-Irradiated Gelatin-Chitosan Bio-Based Composite Film, Physiochemical Features and Release Properties for Packaging Applications. Int. J. Biol. Macromol..

[23] Fathi N., Almasi H., Pirouzifard M.K. (2018). Effect of Ultraviolet Radiation on Morphological and Physicochemical Properties of Sesame Protein Isolate Based Edible Films. Food Hydrocoll..

[24] Schmid M., Held J., Hammann F., Schlemmer D., Noller K. (2015). Effect of UV-Radiation on the Packaging-Related Properties of Whey Protein Isolate Based Films and Coatings. Packag. Technol. Sci..

[25] Michael F.M., Khalid M., Walvekar R., Siddiqui H., Balaji A.B. (2018). Surface Modification Techniques of Biodegradable and Biocompatible Polymers. Biodegradable and Biocompatible Polymer Composites.

[26] Romani V.P., Martins V.G., Goddard J.M. (2020). Radical Scavenging Polyethylene Films as Antioxidant Active Packaging Materials. Food Control.

[27] Goddard J.M., Talbert J.N., Hotchkiss J.H. (2007). Covalent Attachment of Lactase to Low-Density Polyethylene Films. J. Food Sci..

[28] Raghavan S., Kristinsson H.G. (2009). ACE-Inhibitory Activity of Tilapia Protein Hydrolysates. Food Chem..

[29] Adler-Nissen J. (1986). Enzymatic Hydrolysis of Food Proteins.

[30] Kessler F., Kühn S., Radtke C., Weibel D.E. (2013). Controlling the Surface Wettability of Poly(Sulfone) Films by UV-Assisted Treatment: Benefits in Relation to Plasma Treatment. Polym. Int..

[31] Kessler F., Steffens D., Lando G.A., Pranke P., Weibel D.E. (2014). Wettability and Cell Spreading Enhancement in Poly(Sulfone) and Polyurethane Surfaces by UV-Assisted Treatment for Tissue Engineering Purposes. Tissue Eng. Regen. Med..

[32] (2002). ASTM Standard Test Methods for Tensile Properties of Thin Plastic Sheeting.

[33] Silva Filipini G., Romani V.P., Guimarães Martins V. (2021). Blending Collagen, Methylcellulose, and Whey Protein in Films as a Greener Alternative for Food Packaging: Physicochemical and Biodegradable Properties. Packag. Technol. Sci..

[34] ASTM (2000). ASTM E96: Standard Test Methods for Water Vapor Transmission of Materials. Annu. B. ASTM Stand..

[35] Bonilla J., Talón E., Atarés L., Vargas M., Chiralt A. (2013). Effect of the Incorporation of Antioxidants on Physicochemical and Antioxidant Properties of Wheat Starch-Chitosan Films. J. Food Eng..

[36] Bitencourt C.M., Fávaro-Trindade C.S., Sobral P.J.A., Carvalho R.A. (2014). Gelatin-Based Films Additivated with Curcuma Ethanol Extract: Antioxidant Activity and Physical Properties of Films. Food Hydrocoll..

[37] Canabady-Rochelle L.L.S., Harscoat-Schiavo C., Kessler V., Aymes A., Fournier F., Girardet J.M. (2015). Determination of Reducing Power and Metal Chelating Ability of Antioxidant Peptides: Revisited Methods. Food Chem..

[38] Bligh E.G., Dyer W.J. (1959). A Rapid Method of Total Lipid Extraction and Purification. Can. J. Biochem. Physiol..

[39] AOCS (1998). AOCS Official Methods and Recommended Practices of the American Oil Chemists Society.

[40] Song N.B., Lee J.H., Al Mijan M., Song K. (2014). Bin Development of a Chicken Feather Protein Film Containing Clove Oil and Its Application in Smoked Salmon Packaging. LWT Food Sci. Technol..

[41] Gennadios A., Rhim J.-W., Handa A., Weller C.L., Hanna M.A. (1998). Ultraviolet Radiation Affects Physical and Molecular Properties of Soy Protein Films. J. Food Sci..

[42] Khan B., Khan Niazi M.B., Jahan Z., Farooq W., Naqvi S.R., Ali M., Ahmed I., Hussain A. (2019). Effect of Ultra-Violet Cross-Linking on the Properties of Boric Acid and Glycerol Co-Plasticized Thermoplastic Starch Films. Food Packag. Shelf Life.

[43] Ustunol Z., Mert B. (2006). Water Solubility, Mechanical, Barrier, and Thermal Properties of Cross-Linked Whey Protein Isolate-Based Films. J. Food Sci..

[44] Díaz O., Candia D., Cobos Á. (2017). Whey Protein Film Properties as Affected by Ultraviolet Treatment under Alkaline Conditions. Int. Dairy J..

[45] Rhim J.W., Gennadios A., Fu D., Weller C.L., Hanna M.A. (1999). Properties of Ultraviolet Irradiated Protein Films. LWT Food Sci. Technol..

[46] Pankaj S.K., Bueno-Ferrer C., Misra N.N., Bourke P., Cullen P.J. (2014). Zein Film: Effects of Dielectric Barrier Discharge Atmospheric Cold Plasma. J. Appl. Polym. Sci..

[47] Detduangchan N., Wittaya T. (2011). Effect of UV-Treatment on Properties of Biodegradable Film From Rice Starch. World Acad. Sci. Eng. Technol..

[48] Thakhiew W., Devahastin S., Soponronnarit S. (2013). Physical and Mechanical Properties of Chitosan Films as Affected by Drying Methods and Addition of Antimicrobial Agent. J. Food Eng..

[49] Martin I., Goormaghtigh E., Ruysschaert J.-M. (2003). Attenuated Total Reflection IR Spectroscopy as a Tool to Investigate the Orientation and Tertiary Structure Changes in Fusion Proteins. Biochim. Biophys. Acta Biomembr..

[50] Tatulian S.A., Kleinschmidt J.H. (2013). Lipid-Protein Interactions. Lipid-Protein Interactions.

[51] Rostami A.H., Motamedzadegan A., Hosseini S.E., Rezaei M., Kamali A. (2017). Evaluation of Plasticizing and Antioxidant Properties of Silver Carp Protein Hydrolysates in Fish Gelatin Film. J. Aquat. Food Prod. Technol..

[52] Giménez B., Alemán A., Montero P., Gómez-Guillén M.C. (2009). Antioxidant and Functional Properties of Gelatin Hydrolysates Obtained from Skin of Sole and Squid. Food Chem..

[53] Halal S.L.M.E., Zavareze E.D.R., Da Rocha M., Pinto V.Z., Nunes M.R., Luvielmo M.D.M., Prentice C. (2016). Films Based on Protein Isolated from Croaker (*Micropogonias furnieri*) and Palm Oil. J. Sci. Food Agric..

[54] Wiriyaphan C., Chitsomboon B., Yongsawadigul J. (2012). Antioxidant Activity of Protein Hydrolysates Derived from Threadfin Bream Surimi Byproducts. Food Chem..

[55] Bhatia S., Shah Y.A., Al-Harrasi A., Alhadhrami A.S., ALHashmi D.S.H., Jawad M., Dıblan S., Al Dawery S.K.H., Esatbeyoglu T., Anwer M.K. (2024). Characterization of Biodegradable Films Based on Guar Gum and Calcium Caseinate Incorporated with Clary Sage Oil: Rheological, Physicochemical, Antioxidant, and Antimicrobial Properties. J. Agric. Food Res..

[56] Akhtar H.M.S., Zhao Y., Li L., Shi Q. (2024). Novel Active Composite Films Based on Carboxymethyl Cellulose and Sodium Alginate Incorporated with Phycocyanin: Physico-Chemical, Microstructural and Antioxidant Properties. Food Hydrocoll..

[57] Bhatia S., Al-Harrasi A., Shah Y.A., Saif Alrasbi A.N., Jawad M., Koca E., Aydemir L.Y., Alamoudi J.A., Almoshari Y., Mohan S. (2024). Structural, Mechanical, Barrier and Antioxidant Properties of Pectin and Xanthan Gum Edible Films Loaded with Grapefruit Essential Oil. Heliyon.

[58] Wei M., Shan M., Zhang L., Chen N., Tie H., Xiao Y., Li Z. (2024). Preparation of Gelatin/ĸ-Carrageenan Active Films through Procyanidins Crosslinking: Physicochemical, Structural, Antioxidant and Controlled Release Properties. Food Hydrocoll..

[59] Barbosa-Pereira L., Aurrekoetxea G.P., Angulo I., Paseiro-Losada P., Cruz J.M. (2014). Development of New Active Packaging Films Coated with Natural Phenolic Compounds to Improve the Oxidative Stability of Beef. Meat Sci..

[60] Thakur R., Wickramarachchi S., Pal K., Sarkar P. (2024). Gelatin/Chitosan-Lactate/Curcuma Hydroethanolic Extract-Based Antimicrobial Films: Preparation, Characterization, and Application on Chicken Meat. Food Hydrocoll..

[61] Insausti K., Beriain M.J., Purroy A., Alberti P., Gorraiz C., Alzueta M.J. (2001). Shelf Life of Beef from Local Spanish Cattle Breeds Stored under Modified Atmosphere. Meat Sci..

[62] Alemán A., Pérez-Santín E., Bordenave-Juchereau S., Arnaudin I., Gómez-Guillén M.C., Montero P. (2011). Squid Gelatin Hydrolysates with Antihypertensive, Anticancer and Antioxidant Activity. Food Res. Int..

