# Supplementary material for: UV Radiation and Protein Hydrolysates in Bio-Based Films: Impacts on Properties and Italian Salami Preservation
Source: Antioxidants (Basel). 2024 Apr 26;13(5):517. doi: 10.3390/antiox13050517 (PMC11117594; doi:10.3390/antiox13050517)
Supplement: Supplementary file 1 [file antioxidants-13-00517-s001.zip › antioxidants-2959534-supplementary.pdf]

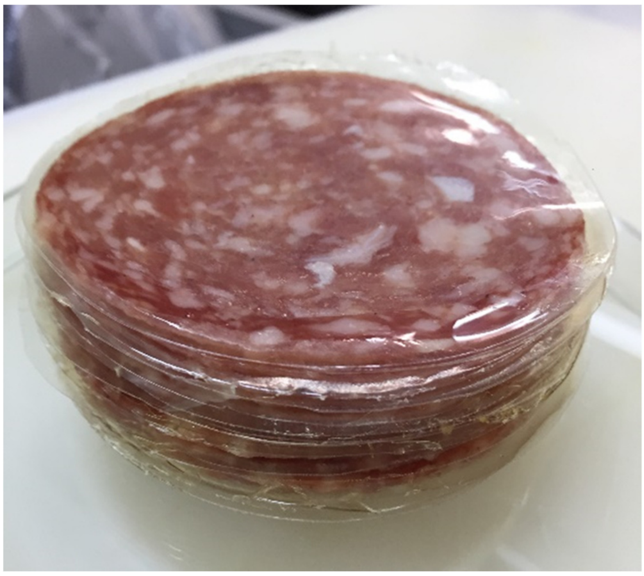

Figure S1. Lipid oxidation assay of sliced Italian salami containing films.

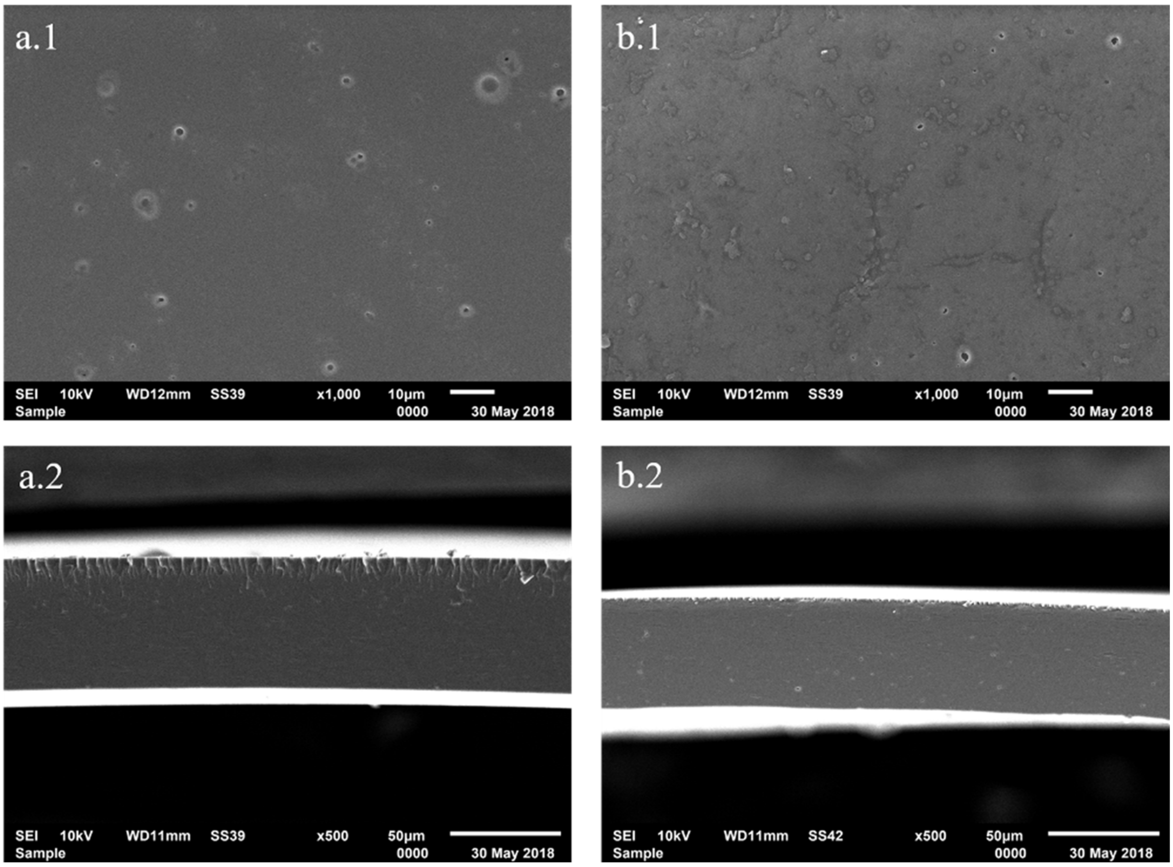

Figure S2. Effects of UV-light treatment on surface morphology (1) and cross-section area (2) of starch/protein films: a) control film and b) UV-treated film (1 min) coated with protein hydrolysates.

Determination of protein hydrolysates amino acid composition

The amino acid composition of proteins hydrolysate was determined similar to Alemán et al. [1] with modifications. Briefly, the protein hydrolysates were dissolved in distilled water (5 mg/mL). An aliquot of 20 mL was dried and hydrolyzed in vacuum-sealed glass tubes at 110 °C for 24 h at constant boiling with 6 N HCl, containing 0.1% phenol. Then, protein hydrolysates were vacuum-dried again, dissolved in application buffer and injected into a Biochrom 20 amino acid analyzer (Pharmacia). Norleucine (Sigma-Aldrich, United States) was used as internal standard.

Film	T_g (ºC)	T_m (ºC)	ΔH (J/g)
Control	53.31	85.71	146.27
UV-treated (1 min)	46.18	63.26	105.87

Table S1. DSC characterization of control and UV-radiated films coated with protein hydrolysates; Tg: Glass transition temperature; Tm: Melting temperature; ΔH: Enthalpy.

Amino acids	Composition (mg/g protein)
Alanine (Ala)	58.40 ± 0.04
Arginine (Arg)	61.01 ± 0.46
Aspartic acid (Asp)	136.93 ± 0.38
Cysteine (Cys)	4.16 ± 0.00
Glutamic acid (Glu)	188.60 ± 1.3
Glycine (Gly)	35.87 ± 0.28
Histidine (His)	24.66 ± 0.14
Isoleucine (Ile)	31.00 ± 0.09
Leucine (Leu)	83.96 ± 0.19
Lysine (Lys)	96.07 ± 0.36
Methionine (Met)	41.68 ± 0.48
Phenylalanine (Phe)	38.67 ± 0.01
Proline (Pro)	33.37 ± 0.46
Serine (Ser)	46.04 ± 0.40
Threonine (Thr)	45.38 ± 0.62

Tyrosine (Tyr)	38.32 ± 0.11
Valine (Val)	35.87 ± 0.02
HAA	365.44 ± 0.34
AAA	77.00 ± 0.11
NCAA	325.53 ± 1.75
PCAA	181.74 ± 0.68
EAA	390.23 ± 0.28

Table S2. Amino acid composition of protein hydrolysates. HAA: hydrophobic amino acids (alanine, valine, isoleucine, leucine, tyrosine, phenylalanine, proline, methionine, and cysteine); AAA: aromatic amino acids (phenylalanine, tryptophan and tyrosine); NCAA: negatively charged amino acids (aspartic acid, glutamic acid); PCAA: positively charged amino acids (arginine, histidine, lysine); EEA: essential amino acids (phenylalanine, valine, threonine, isoleucine, methionine, histidine, leucine and lysine).

Reference

1. Alemán, A.; Pérez-Santín, E.; Bordenave-Juchereau, S.; Arnaudin, I.; Gómez-Guillén, M.C.; Montero, P. Squid Gelatin Hydrolysates with Antihypertensive, Anticancer and Antioxidant Activity. *Food Res. Int.* **2011**, *44*, 1044–1051, doi:10.1016/j.foodres.2011.03.010.
